# Massively parallel genomic perturbations with multi-target CRISPR interrogates Cas9 activity and DNA repair at endogenous sites

**DOI:** 10.1038/s41556-022-00975-z

**Published:** 2022-09-05

**Authors:** Roger S. Zou, Alberto Marin-Gonzalez, Yang Liu, Hans B. Liu, Leo Shen, Rachel K. Dveirin, Jay X. J. Luo, Reza Kalhor, Taekjip Ha

**Affiliations:** 1 Department of Biomedical Engineering, Johns Hopkins University School of Medicine, Baltimore, MD USA; 2 Department of Biophysics and Biophysical Chemistry, Johns Hopkins University School of Medicine, Baltimore, MD USA; 3 Department of Biophysics, Johns Hopkins University, Baltimore, MD USA; 4 Howard Hughes Medical Institute, Baltimore, MD USA

**Keywords:** CRISPR-Cas9 genome editing, DNA damage response, Double-strand DNA breaks

## Abstract

Here we present an approach that combines a clustered regularly interspaced short palindromic repeats (CRISPR) system that simultaneously targets hundreds of epigenetically diverse endogenous genomic sites with high-throughput sequencing to measure Cas9 dynamics and cellular responses at scale. This massive multiplexing of CRISPR is enabled by means of multi-target guide RNAs (mgRNAs), degenerate guide RNAs that direct Cas9 to a pre-determined number of well-mapped sites. mgRNAs uncovered generalizable insights into Cas9 binding and cleavage, revealing rapid post-cleavage Cas9 departure and repair factor loading at protospacer adjacent motif-proximal genomic DNA. Moreover, by bypassing confounding effects from guide RNA sequence, mgRNAs unveiled that Cas9 binding is enhanced at chromatin-accessible regions, and cleavage by bound Cas9 is more efficient near transcribed regions. Combined with light-mediated activation and deactivation of Cas9 activity, mgRNAs further enabled high-throughput study of the cellular response to double-strand breaks with high temporal resolution, revealing the presence, extent (under 2 kb) and kinetics (~1 h) of reversible DNA damage-induced chromatin decompaction. Altogether, this work establishes mgRNAs as a generalizable platform for multiplexing CRISPR and advances our understanding of intracellular Cas9 activity and the DNA damage response at endogenous loci.

## Abstract (web summary)

Zou et al. use a single multi-target guide RNA to direct Cas9 to a high number of loci mapped by high-throughput short-read sequencing. This multi-target CRISPR system allows detailed studies of genome editing and DNA repair.

## Main

Clustered regularly interspaced short palindromic repeats (CRISPR)–Cas nucleases such as *Streptococcus pyogenes* Cas9 have revolutionized biomedicine through genome manipulation^1^. For genome editing, Cas9 binds to DNA complementary to its guide RNA (gRNA), induces a double-strand break (DSB), then initiates DNA damage responses (DDRs) that repair and potentially modify the DNA sequence^2^. Although several studies have shed light on different stages of this process^3–9^, many aspects of intracellular Cas9 behaviour and ensuing DDR remain incompletely characterized. For instance, how Cas9 departs from genomic DNA after cleavage is unclear^10–12^, and how genomic context combines with mismatch levels to dictate Cas9 binding and cleavage requires more characterization. The cellular response to Cas9-induced DNA damage also warrants further study^6,13,14^, in particular, how damage response factors and chromatin interact with genomic DNA cleaved by Cas9 (refs. ^6,13,15^).

Better understanding of these CRISPR-associated processes would further mature CRISPR technologies and inspire future tools and applications^14,16–18^. However, current approaches have been limited to few target sites, in vitro measurements, reporter systems or expressed libraries of gRNAs. Limited target positions preclude exploring heterogeneity at different genomic locations to extract generalizable conclusions^19,20^, while in vitro measurements fail to capture the complex chromatin context and are not always generalizable to inside cells^11,12,21^. Reporter systems may not reflect endogenous phenotypes^8,9^, and expressed gRNA libraries introduce variability between individual gRNAs, thus obscuring readouts on relative Cas9 activity at different target sites^4,8^.

**Fig. 1:**
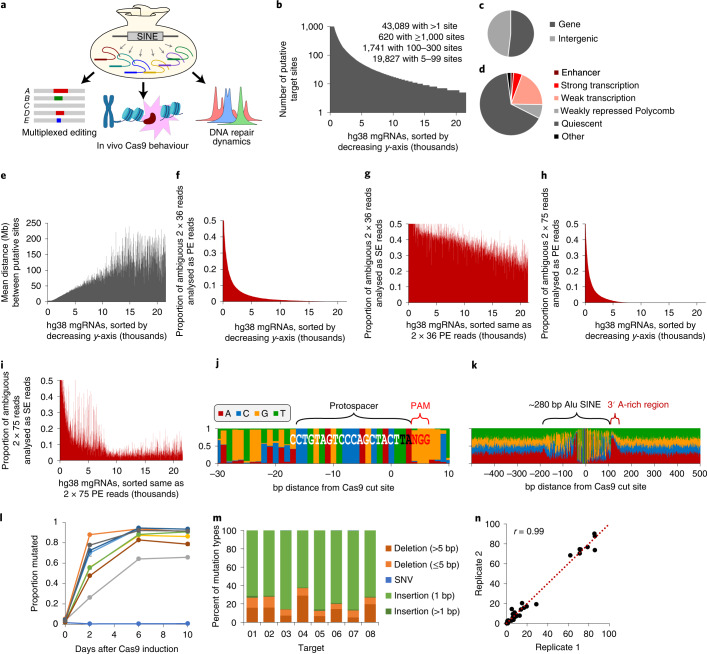
Initial characterization of mgRNAs. **a**, Schematic of mgRNA discovery and applications. **b**, Plot of the thousands of unique target sequences identified in silico, sorted along the *x*-axis by the number of putative on-target sites in the hg38 human genome (*y*-axis). A maximum of 1,000 on-target sites were evaluated for each gRNA (even if the total was higher) to maintain efficient computation. **c**,**d**, RefSeq (**c**) and ChromHMM (**d**) classification of target sequences. In **d**, only labels with ≥1% representation are directly displayed; otherwise they are grouped under ‘Other’. **e**, For the unique target sequences shown in **b**, we plotted the mean distance between adjacent putative on-target sites on the same chromosome. The order of sequences along the *x*-axis is the same as in **b**. **f**, Using bowtie2, we determined the proportion of ambiguous reads from simulated PE 2 × 36 bp ChIP–seq reads at on-target sites for each target sequence. Target sequences (*x*-axis) are sorted by decreasing proportion of ambiguous reads (*y*-axis). **g**, Same as **f**, but interpreted as two SE 36 bp ChIP–seq reads. **h**,**i**, Same as **f** and **g**, respectively, but using 75 bp ChIP–seq reads, instead of 36 bp. **j**,**k**, Nucleotide composition at each position in a window of 40 bp (**j**) or 1 kb (**k**) around all Cas9 cut sites for a select mgRNA. The *x*-axis represents the base-pair distance from the Cas9 cut site at *x* = 0 (fourth nucleotide from PAM). Protospacer, PAM, the ~280 bp SINE and its 3′ A-rich region are annotated. The gRNA sequence is written in white and black font and the PAM in red. **l**, Mutation rate of ten-target mgRNA. HeLa cells with Dox-inducible Cas9 were transduced with a ten-target mgRNA and grown in presence of Dox. Cells were collected at different timepoints (0, 2, 6 and 10 days). The genomic DNA was extracted, PCR amplified and sequenced at the Cas9 target sites (for details, see Methods). Data points are the average of two biological replicates. **m**, Day-10 samples from **i** were analysed to determine the mutational signatures resulting from Cas9 cleavage at each target (8/10) that showed detectable indels. Mutations were classified into five categories: long deletions (longer than 5 bp), short deletions (5 bp or less), SNVs (that is, base change), 1 bp insertions, and insertions longer than 1 bp. Only the mutated protospacers were considered for the analysis. Data points are the average of two biological replicates. **n**, Reproducibility analysis of **m**. Each point represents the percentage of a particular mutation type for a given target (akin to the bars in **m**, but with replicates analysed separately). Data points were fitted to a linear function (dotted line) and their Pearson correlation coefficient was computed, yielding *r* = 0.99. Source numerical data are available in source data. Source data

In this Technical Report, we present an approach whereby a single, multi-target gRNA (mgRNA) directs Cas9 to simultaneously target over a hundred endogenous positions genome-wide that are well mapped by high-throughput short-read sequencing. This technique enabled interrogation of Cas9 activity and the ensuing DDRs at endogenous sites at scale. Using mgRNAs, we made discoveries on the dynamics of Cas9 binding and post-cleavage release, the effects of chromatin context on Cas9 activity, and chromatin dynamics during the cycle of DNA damage to repair (Fig. 1a). Our findings establish multi-target CRISPR as a generalizable platform for advancing our understanding of CRISPR-based genome manipulation and cellular DNA damage and repair.

## Results

### Design, discovery and characterization of mgRNAs

To discover Cas9 gRNA sequences with multiple target positions in the genome, we searched for 20 bp sequences adjacent to a Cas9 protospacer adjacent motif (PAM) in the human genome with up to three mismatches from a 280 bp short interspersed nuclear element (SINE)^22^. Over 40,000 20 bp sequences were found, each targeting between 2 and over 1,000 putative on-target sites (Fig. 1b). The target sites are located throughout the genome, exhibit balanced representation between gene bodies and intergenic regions, and represent multiple epigenetic states^23^ (Fig. 1c–e).

**Extended Data Fig. 1:**
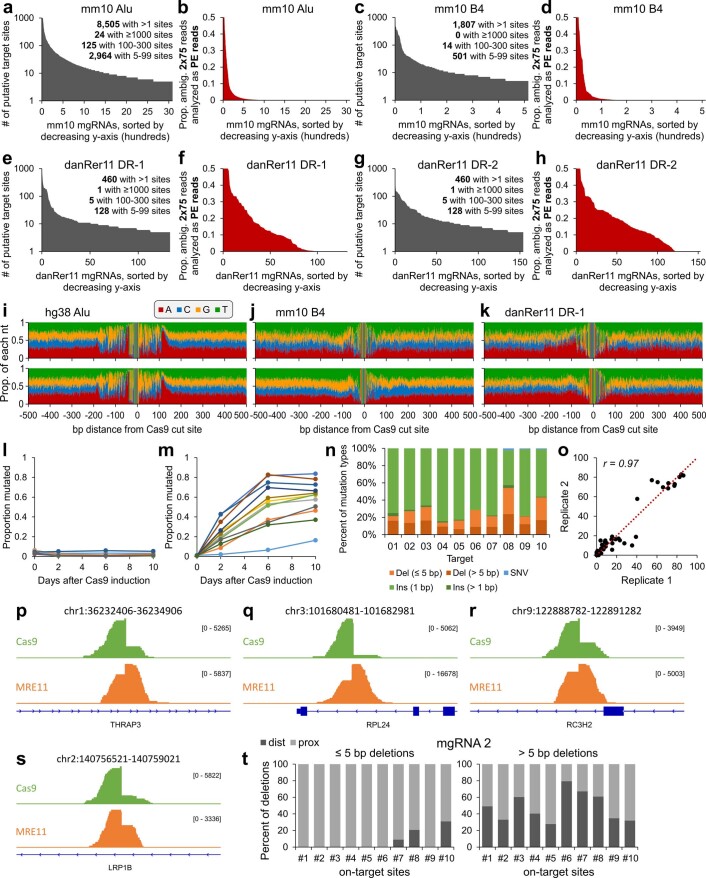
Additional *in silico* and experimental characterization of multi-target gRNAs. **a**,**b**, Same as Fig. 1b and Fig. 1h, respectively, but for the Alu SINE in the mouse mm10 genome. **c**,**d**, Same as Fig. 1b and Fig. 1h, respectively, but for the B4 SINE in the mouse mm10 genome. **e**,**f**, Same as Fig. 1b and Fig. 1h, respectively, but for the DR-1 SINE in the zebrafish danRer11 genome. **g**,**h**, Same as Fig. 1b and Fig. 1h, respectively, but for the DR-2 SINE in the zebrafish danRer11 genome. **i**-**k**, Same as Fig. 1k, but for two different mgRNAs from the (**i**) Alu SINE in the human hg38 genome, (**j**) B4 SINE in the mouse mm10 genome, and (**k**) DR-1 SINE in the zebrafish danRer11 genome. **l**, No-dox control mutation curve. Dox-inducible Cas9 cells genomically integrated with a 10-target mgRNA (same cells as in Fig. 1l) were grown and passaged without dox exposure. Cells were harvested at different time points (0, 2, 6 and 10 days). **m**, Mutation curve of 20-target mgRNA in HeLa cells with dox-inducible Cas9. Figure details are the same as Fig. 1l. **n**, Mutation signatures for 10-day samples from **m**. Figure details are the same as in Fig. 1m. **o**, Same reproducibility analysis and figure details as in Fig. 1n but using the mgRNA from **m**. **p**-**s**, For all 4 target sites, the PAM-distal side is oriented on the left. (**p**-**r**) The first three examples exhibit Cas9 PAM-distal bias and MRE11 PAM-proximal bias. However, the last example (**s**) in chr2:140756521-140759021 illustrates an exception where both Cas9 and MRE11 exhibit PAM-distal bias. **t**, Same as Fig. 3j, but with the 20-target mgRNA 2. Source numerical data are available in source data. Source data

We then evaluated whether the targeted regions can be uniquely distinguished with high-throughput short-read sequencing. We generated simulated Illumina-style paired-end (PE) 2 × 36 bp reads at all target sites for each gRNA with 5–300 target sites, then determined the number of genome-wide alignments for each read using bowtie2 (ref. ^24^). For the majority of gRNAs, only a small minority of reads had ambiguous alignments, that is, more than one alignment with the same ‘best’ bowtie2 alignment score (Fig. 1f). As expected, treating the PE 2 × 36 bp reads as single-end (SE) reads increased the percentage of reads with ambiguous alignments (Fig. 1g), whereas increasing the number of sequenced base pairs to 75 at each end (that is, PE 2 × 75 bp) reduced this percentage (Fig. 1h, i). We then aligned the sequence around each expected on-target site for a gRNA with under 1% ambiguous alignments. The nucleotide composition at each position in a 40 bp window confirmed the expected Cas9 protospacer (Fig. 1j). Expanding to a 1 kb window confirmed features of the Alu SINE, such as its 280 bp approximate length and A-rich 3′ end^22^ (Fig. 1k). The sequences beyond 150–200 bp from the cut sites were evenly distributed between the four nucleotides and probably correspond to regions that can be uniquely mapped by sequencing. PE sequencing reads can therefore be uniquely mapped given the sequence diversity even within the short repetitive element and the high probability of at least one DNA end being positioned outside the element. We replicated the same analysis using a mouse and a zebrafish genome and different SINEs^22^ (Extended Data Fig. 1a–k). Together, our computational pipeline robustly identified diverse candidate mgRNAs across different species.

### Experimental validation of mgRNAs

We validated the activity of mgRNAs by measuring genome editing outcomes (insertions and deletions, or indels) at mgRNA targeted sites. Cas9/mgRNA with ten predicted target sites active in HeLa cells over 10 days revealed robust indel generation at eight out of the ten sites (Fig. 1l and Extended Data Fig. 1l). The mutation identity was predominantly one-nucleotide insertions, consistent with the repair profiles of Cas9-generated DSBs^8,9,25–27^ (Fig. 1m). Similar results were obtained with a different mgRNA (Extended Data Fig. 1m,n), and mutation distributions showed high reproducibility between biological replicates (Fig. 1n and Extended Data Fig. 1o). Together, these results demonstrate efficient intracellular activity with mgRNAs.

**Fig. 2:**
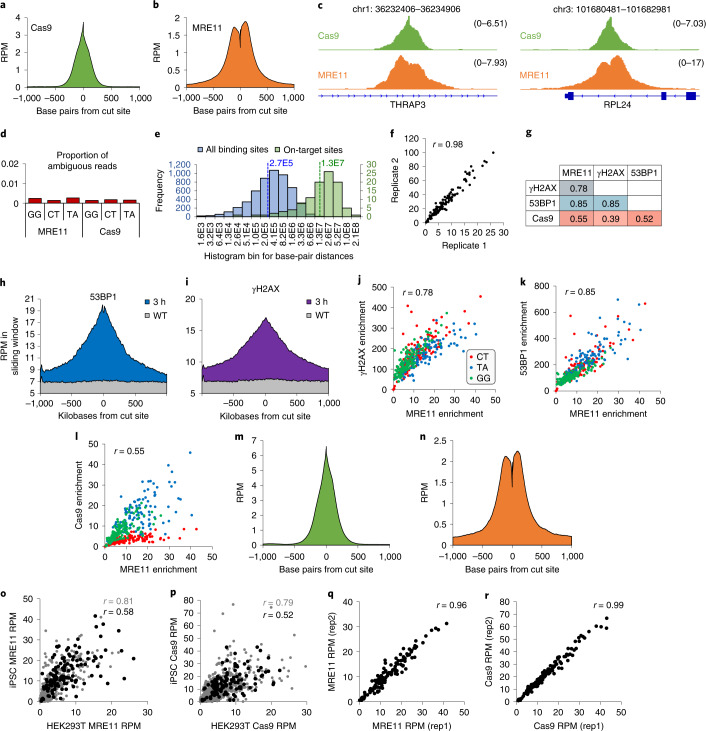
Validation of mgRNAs using ChIP–seq. **a**,**b**, Average profiles of Cas9 (**a**) and MRE11 (**b**) enrichment in a 2,000 bp window centred at the cut site, across all on-target sites. **c**, Distribution of PE Cas9 and MRE11 ChIP–seq read fragments that span the cut, at two particular cut sites. Coordinates in hg38 are listed in the title of each panel. Numerical value of enrichment is reported as reads per million (RPM). **d**, Proportion of ambiguous reads from measured MRE11 and Cas9 ChIP–seq reads with the three target sequences ‘GG’, ‘CT’ and ‘TA’. Reads at all discovered macs2 peaks were used for analysis, which include both on-target and potential off-target sites. **e**, Histogram of distances between all adjacent macs2 detected Cas9 binding sites (blue; median distance of 265 kb represented by blue dashed line), and between all adjacent on-target sites (green; median distance of 13.2 Mb represented by green dashed line). **f**, Correlation between two biological replicates of MRE11 ChIP–seq. Both axes have units of RPM enrichment in a 2.5 kb window centred at each cut site. **g**, All possible correlations between MRE11, Cas9, γH2AX and 53BP1 ChIP–seq enrichment measured in a specific window centred at all on-target sites. A 2,500 bp window was used for MRE11; 1,500 bp for Cas9; and 20 kb for γH2AX and 53BP1. **h**,**i**, Average profiles of 53BP1 (**h**) and γH2AX (**i**) enrichment in a 2 Mb window centred at the cut sites. **j**–**l**, Plots of relationships between MRE11 and γH2AX (**j**), MRE11 and 53BP1 (**k**), and MRE11 and Cas9 (**l**) at each target site of the three different target sequences (‘CT’, red; ‘TA’, blue; ‘GG’, green). **m**,**n**, Average profile of MRE11 (**m**) and Cas9 (**n**) enrichment in a 2,000 bp window centred at all cut sites in WTC-11 iPSCs. **o**,**p**, Plot of MRE11 (**o**) and Cas9 (**p**) ChIP–seq enrichment around all on-target sites (black) and all binding sites (on-target and off-target; grey), between HEK293T cells and iPSCs. Pearson correlation coefficient displayed on graph. **q**,**r**, Plot of MRE11 (**m**) and Cas9 (**n**) ChIP–seq enrichment around all target sites, between iPSC biological replicates. Source numerical data are available in source data. Source data

To interrogate Cas9 binding and recruitment of DNA repair factors in a high-throughput manner, we tested three mgRNAs (‘CT’, ‘GG’ and ‘TA’) with 145, 126 and 117 on-target sites, respectively. We electroporated Cas9 protein pre-assembled with mgRNA into HEK293T cells, followed 3 h later by chromatin immunoprecipitation with sequencing (ChIP–seq) for Cas9 and an early DDR protein, MRE11 (refs. ^4,5,13,28^). ChIP–seq profiles averaged across all on-target sites revealed high enrichment with shapes consistent with previous literature^4,5,13,28^ (Fig. 2a–c). Cas9 on- and off-target sites were called using MACS2 software^29^, and showed less than 0.3% of sequencing reads with ambiguous alignments (Fig. 2d), verifying that ChIP–seq accurately quantified enrichment at sites targeted by these mgRNAs. Median distances between adjacent Cas9 binding sites and adjacent on-target sites were both large, at 270 kb and 13 Mb, respectively **(**Fig. 2e). MRE11 enrichment was highly correlated between biological replicates (Fig. 2f) and with other DNA repair markers such as 53BP1 and phosphorylated H2AX (γH2AX)^30^ (Fig. 2g–k). In contrast, correlations between Cas9 and DNA repair factors were weaker and dependent on gRNA sequence (Fig. 2g,l). MRE11 and Cas9 ChIP–seq after mgRNA delivery was also performed in induced pluripotent stem cells (iPSCs) (Fig. 2m,n). ChIP–seq enrichments at target sites were only moderately correlated between iPSCs and HEK293T cells (Fig. 2o,p) despite high correlation between biological replicates (Fig. 2q,r). Altogether, these results demonstrate multiplexed Cas9 activity and robust ChIP–seq readout for Cas9 and DNA repair factors at endogenous sites targeted by mgRNAs.

### Cas9 binding and cleavage mechanics at endogenous loci

**Fig. 3:**
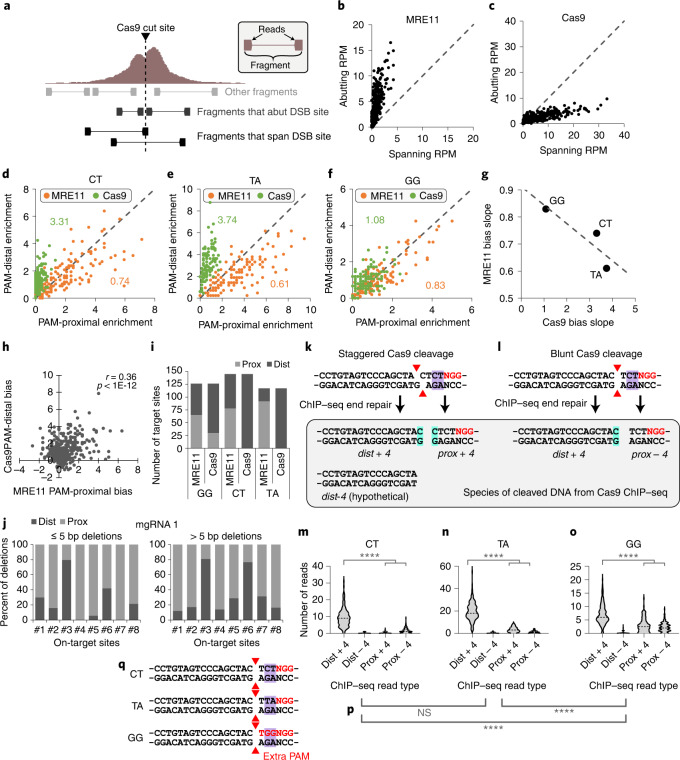
Analysis of Cas9 cleavage features from ChIP–seq data. **a**, Conceptual schematic of ChIP–seq analysis for PE reads, distinguishing between read fragments that abut or span the DSB site. Reads abut the DSB site if they fall within 5 bp from the cut site. **b**,**c**, Number of PE reads (RPM) that either span or abut each target site for MRE11 (**b**) and Cas9 (**c**) ChIP–seq. **d**–**f**, For each ‘CT’ (**d**), ‘TA’ (**e**) and ‘GG’ (**f**) mgRNA on-target site, RPM on the PAM-proximal versus PAM-distal side for MRE11 (orange) versus Cas9 (green) ChIP–seq 3 h after Cas9 delivery. The numbers indicate linear regression slopes. **g**, MRE11 and Cas9 slopes from **d**–**f** are inversely correlated. **h**, For each target site, we plotted the MRE11 PAM-proximal bias versus Cas9 PAM-distal bias. PAM-proximal bias is defined as RPM on the PAM-proximal side minus RPM on the PAM-distal side, and vice versa for PAM-distal bias. Correlation was determined using Pearson correlation with its *P* value. **i**, For MRE11 and Cas9 ChIP–seq using the ‘GG’, ‘CT’ or ‘TA’ mgRNAs, we plot the number of on-target sites with PAM-proximal (‘prox’, light grey) or PAM-distal (‘dist’, dark grey), bias. **j**, For cells exposed to 10 days of Cas9 with mgRNA from in Fig. 1l, we determined all possible deletions from high-throughput amplicon sequencing data at select on-target sites (1, 2, …, on *x*-axis). For deletions ≤5 bp or >5 bp, we determined whether the deletion occurs more on the PAM-proximal or PAM-distal side. **k**,**l**, Schematic of Cas9 cleavage scenarios for the ‘CT’ target sequence. Two possibilities for Cas9 cleavage (staggered versus blunt) are displayed, with red triangles annotating the cleavage position at each DNA strand. ChIP–seq end repair fills in nucleotides at the 3′ end, resulting in three possible read species: *dist* + *4*: immediately PAM-distal, containing fourth nucleotide from PAM (+4 nucleotide); *prox* + *4*: immediately PAM-proximal containing +4 nucleotide; *prox* − *4*: immediately PAM-proximal lacking +4 nucleotide. A fourth, hypothetical species is included for completeness: *dist* − *4*: immediately PAM-distal lacking +4 nucleotide. The +4 nucleotide is highlighted in cyan. **m**–**o**, Violin plot of the number of reads, for each Cas9 target site, categorized by the four read types (*dist* + *4*, *dist* − *4*, *prox* + *4* and *prox* − *4*) described above. Comparison between *dist* + *4* and sum of *prox* + *4* and *prox* − *4* using two-sided unadjusted Student’s *t*-test. ****P < 0.0001. *P* values are: 3 × 10^−80^, 3 × 10^−57^ and 3 × 10^−16^ for CT, TA and GG, respectively. **p**, Two-sided unadjusted Student’s *t*-test of significance for the number of PAM-proximal reads (*prox* + *4* + *prox* − *4*) between different gRNA sequences. NS, not significant, ****P < 0.0001. *P* values from left to right are: 0.35, 2.6 × 10^−22^ and 6.6 × 10^−19^. **q**, Schematic of the ‘CT’, ‘TA’ and ‘GG’ sequences, which differ only in the most PAM-proximal two nucleotides (highlighted in purple). The ‘GG’ target sequence has an extra PAM. ‘NGG’ PAM(s) are labelled in red. The blunt-end cleavage possibility is displayed with red triangles annotating the cleavage position. Source numerical data are available in source data. Source data

Characterizing how Cas9 interacts with genomic DNA is important to better understand Cas9 genome editing^5,10,28^. For example, how Cas9 departs from genomic DNA after cleavage is unclear; RNA polymerase^31^ and histone chaperone FACT^12^ have both been proposed to evict Cas9, but direct evidence inside cells is lacking. To dissect these dynamics in a highly multiplexed fashion while controlling for the target sequence, we exposed HEK293T cells to ‘GG’, ‘CT’ or ‘TA’ mgRNAs for 3 h, and categorized the resulting Cas9 and MRE11 ChIP–seq reads as either spanning or abutting the cut site, corresponding to protein-associated DNA fragments that are either intact or cleaved by Cas9, respectively^13,28^ (Fig. 3a). MRE11 ChIP–seq reads predominantly abutted the cut sites (Fig. 3b), consistent with MRE11 loading on cleaved DNA^13,28^, whereas Cas9 ChIP–seq reads predominantly spanned the cut sites (Fig. 3c), consistent with Cas9 residing on the target before cleavage and departing quickly thereafter. Of the reads that abut each cut site, MRE11 exhibited enrichment bias for the PAM-proximal side of the cut for most target sites, while Cas9 showed bias for the PAM-distal side (Fig. 3d–f and Extended Data Fig. 1p–r). The extent of PAM-proximal/PAM-distal bias was inversely correlated between MRE11 and Cas9 though not all target sites exhibited this bias (Fig. 3g–i and Extended Data Fig. 1s). These results suggest stable Cas9 binding before cleavage, possibly to check for sequence complementarity, followed by cleavage and rapid release of DNA preferentially from the PAM-proximal side, facilitating MRE11 loading. Consistent with this model, sequencing of indel products showed preferential short deletions at the PAM-proximal (MRE11-resident) side (Fig. 3j and Extended Data Fig. 1t). Preferential Cas9 dissociation from the PAM-proximal side was observed previously, but only for a single target sequence and in vitro^32^. Our results validate this observation in cells and further suggest that Cas9 binding to a cleaved DNA terminus can obfuscate it from MRE11 and the cellular DDR.

To further characterize post-cleavage Cas9 mechanics, we modelled Cas9 ChIP–seq read species derived from DNA fragments bound to Cas9 after either staggered or blunt cleavage^33^. From staggered cleavage, DNA end repair during ChIP–seq library preparation fills in the 3′ end, resulting in presence of the fourth nucleotide (from PAM) at both sides of the cut **(**Fig. 3k**)**. We refer to these ChIP–seq reads on PAM-proximal and PAM-distal sides as *‘prox* + *4*’ and ‘*dist* + *4*, respectively. In contrast, from blunt-end cleavage, only the PAM-distal read contains the fourth nucleotide, that is, ‘*dist* + *4*’, whereas the PAM-proximal read does not, resulting in a ‘*prox* − *4*’ read species (Fig. 3l). ‘*dist* + *4*’ was significantly more enriched than the sum of ‘*prox* + *4*’ and ‘*prox* − *4*’ (*P* < 1 × 10^−15^, Student’s *t*-test), recapitulating clear PAM-distal binding bias (Fig. 3m–o). These results suggest that the 16–17 bp of gRNA to genomic DNA base-pairing interactions at the PAM-distal side of the cut are stronger than the 3–4 bp of base pairing and PAM–Cas9 interactions at the PAM-proximal side. Interestingly, Cas9 with the ‘GG’ gRNA exhibited significantly stronger association with the PAM-proximal side compared with the other two gRNAs (*P* < 1 × 10^−18^, Student’s *t*-test) (Fig. 3p), which we speculate may be due to the additional ‘NGG’ PAM sequence in the first three nucleotides of the protospacer (Fig. 3q).

### Linking Cas9 binding and DNA repair to local epigenetic states

**Fig. 4:**
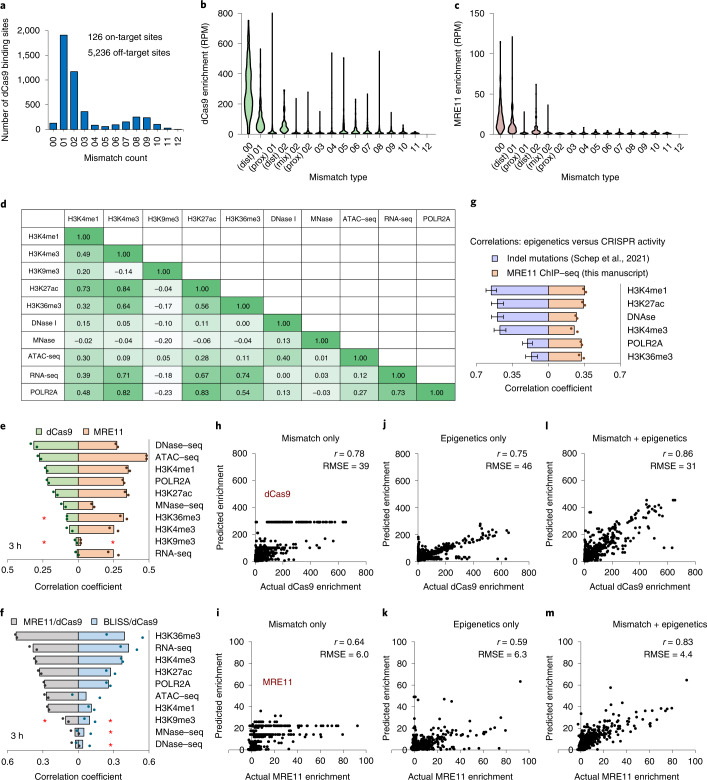
Epigenetic determinants of Cas9 binding and MRE11 recruitment. **a**, Identification of all dCas9/′GG′ gRNA binding sites at 3 h after dCas9 delivery, sorted by mismatch count. ‘00’ corresponds to no mismatches, ‘03’ corresponds to three mismatches, and so on. **b**, Histogram of dCas9 enrichment by mismatch type at 3 h. ‘(dist) 01’ corresponds to one PAM-distal (≥12th nucleotide, counting from PAM) mismatch, ‘(prox) 01’ corresponds to one PAM-proximal (<12th nucleotide) mismatch, ‘(mix) 02’ corresponds to two total mismatches—one PAM-proximal and the other PAM-distal. **c**, Same as **b**, but using MRE11 ChIP–seq after Cas9 delivery. **d**, Correlation coefficients between enrichment of public epigenetic datasets at all on-target sites targeted by the ‘GG’ mgRNA. **e**, Absolute value of correlation coefficients between dCas9 (light green) or MRE11 (light orange) enrichment and epigenetic markers at all on-target sites, evaluated at 3 h after dCas9 or Cas9 delivery, respectively. **f**, Absolute value of correlation coefficients between dCas9-normalized MRE11 enrichment (MRE11/dCas9; grey) or dCas9-normalized BLISS enrichment (BLISS/dCas9; light blue) with epigenetic markers at all on-target sites, evaluated at 3 h after (d)Cas9 delivery. **e**,**f**, The order of epigenetic markers from top to bottom is sorted by the degree of its absolute correlation with dCas9 (**e**) or MRE11/dCas9 enrichment (**f**). The originally negative correlations are marked by red asterisks. **g**, Comparisons of correlations between CRISPR activity (indels in ref. ^7^ versus MRE11 ChIP–seq enrichment in this manuscript) across the same epigenetic markers corresponding to enhancers and transcribed regions. *n* = 4 biologically independent experiments for ref. ^7^ data presented as mean ± standard deviation (s.d.). **h**,**i**, Actual (*x*-axis) versus predicted (*y*-axis) dCas9 (**h**) and MRE11 (**i**) enrichment by a trained random forest regressor on an independent test set. Samples evaluated at 3 h after (d)Cas9 delivery. RMSE is root-mean-square error. **j**,**k**, Same as **h** and **i**, but using epigenetic features (RNA-seq, MNase–seq, ATAC–seq, DNase–seq and H3K4me1, H3K4me3, H3K9me3, H3K27ac, H3K36me3 ChIP–seq) instead of mismatches. **l**,**m**, Same as **h** and **i**, but using both mismatch information and epigenetic features. Source numerical data are available in source data. Source data

**Extended Data Fig. 2:**
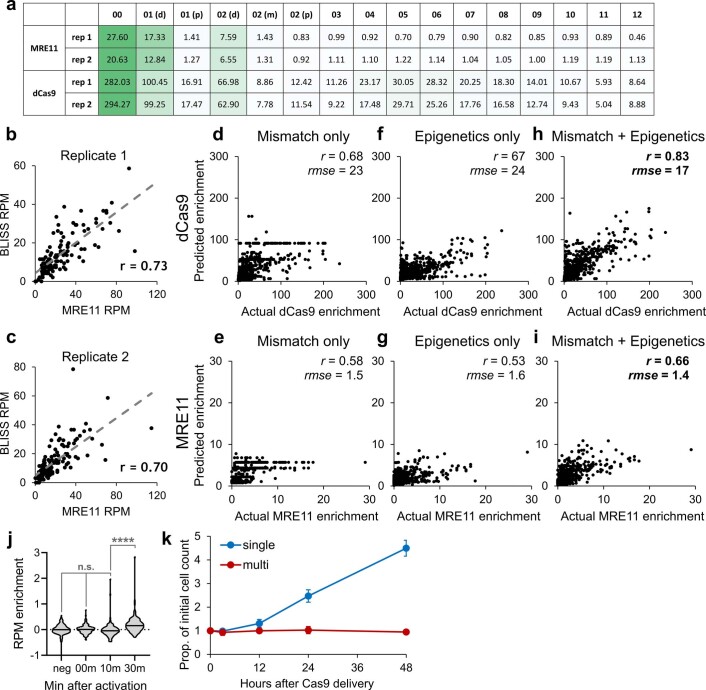
Further analysis and machine learning modeling at 30 min. **a**, Mean quantification of Fig. 4b,c for two biological replicates. **b**,**c**, Two replicates of correlation between MRE11 and BLISS enrichment in 2500 bp windows centered around all cut sites. **d**-**i**, Same as Fig. 4 h-m but for samples evaluated at 30 minutes after (d)Cas9 delivery. **j**, Violin plots of background-subtracted ATAC-seq reads per million (RPM) enrichment at all on-target sites. ‘neg’ indicates Cas9-negative cells. ‘00m’, ‘10m’, and ‘30m’ indicate cells with vfCRISPR Cas9/cgRNA without light exposure, 10 minutes after light exposure, and 30 minutes after light exposure, respectively. Comparison using two-sided unadjusted Student’s t-test. n.s. indicates no significance, **** indicates p<0.0001. p-values from left to right are: 0.64, 0.25, and 6.85E-8. **k**, Quantification of Fig. 7e. n=3 biologically independent experiments, data presented as mean ±1 SD. Source numerical data are available in source data. Source data

Genome editing efficiencies are difficult to predict but are probably influenced by both sequence and epigenetic factors^3,7,21,34^. Epigenetic influences have been challenging to decipher owing to confounding effects of gRNA sequence^19^; mgRNAs are uniquely suited for this task because a common gRNA sequence targets different epigenetic contexts. To characterize Cas9 binding alone, we measured occupancy of (cleavage-deficient) dCas9 using ChIP–seq after mgRNA/dCas9 delivery. For the ‘GG’ mgRNA, we detected 5,236 dCas9 binding sites (Fig. 4a), a number of off-target sites comparable to single-targeting gRNAs^4^. To evaluate Cas9-mediated DNA damage, we measured occupancy of MRE11 after delivery of (cleavage-competent) Cas9. MRE11 was only enriched at sites with two or fewer mismatches whereas some sites exhibited clear dCas9 binding for up to over eight mismatches, and both enrichments were higher if the mismatch resided solely in the PAM-distal region (≥12th position, counting from PAM) (Fig. 4b,c and Extended Data Fig. 2a), consistent with known properties of Cas9 binding and cleavage^5,11,35^. Interestingly, there was high heterogeneity in both dCas9 and MRE11 enrichment even between identical on-target sequences (Fig. 4b,c), probably stemming from epigenetic factors.

To infer the epigenetic state, we obtained ten publicly available genome-wide epigenetic maps from the same cell line^36^ and determined their enrichments in specified windows centred around each Cas9 target site (Fig. 4d). dCas9 enrichment was most strongly correlated with markers of DNA accessibility, as measured by assay for transposase-accessible chromatin using sequencing (ATAC–seq) and DNase I-hypersensitive site sequencing (DNase–seq), consistent with previous reports^4,5,37,38^. In contrast, MRE11 recruitment was correlated with additional chromatin features besides accessibility (Fig. 4e), suggesting that additional epigenetic factors are at play beyond Cas9 binding. To characterize the MRE11 damage response independent of Cas9 binding, we normalized MRE11 signal by dCas9 signal, which yielded the strongest correlation with gene bodies (H3K36me3 and RNA sequencing (RNA-seq)), promoters (H3K4me3 and RNA polymerase II) and enhancers (H3K27ac) (Fig. 4f). This suggests either higher Cas9 cleavage efficiencies or more efficient MRE11 recruitment at these regions, which we can distinguish by directly measuring DSB levels genome-wide using breaks labelling in situ and sequencing (BLISS)^39^. BLISS enrichment was highly correlated with MRE11 (*r* = 0.7) (Extended Data Fig. 2b,c), and the pattern of epigenetic correlation for dCas9-normalized BLISS enrichment (unrepaired DSBs given the same amount of Cas9 binding) mirrored dCas9-normalized MRE11 enrichment (Fig. 4f). These results suggest that identical Cas9 on-target sites bound by the Cas9–gRNA complex are cleaved at different rates. In particular, regions near gene bodies, promoters and enhancers exhibit intrinsically higher cleavage activity by a bound Cas9. Together, improved Cas9 binding at accessible regions, followed by increased Cas9-mediated DNA damage near enhancers, promoters and gene bodies, provides an explanation for previous studies using sgRNAs that report higher genome editing efficiencies at these exact regions (Fig. 4g)^3,5,21,34^.

The biophysical mechanism for improved Cas9 cleavage near transcribed regions requires further investigation. One possible explanation is DNA supercoiling; transcribed regions are known to be negatively supercoiled^40,41^, and single-molecule biophysical studies showed that Cas9 cleaves more efficiently on DNA negatively supercoiled at physiologically relevant levels^42^. Other potential mechanisms include DNA-binding proteins such as RNA polymerase^31^ and the histone chaperone FACT^12^ influencing Cas9 residence on gDNA.

### Prediction of genome editing processes using machine learning

To further explore the determinants of Cas9 binding and DNA damage induction, we trained random forest machine learning models to predict both dCas9 and MRE11 enrichment at all binding locations. From solely mismatch information, dCas9 and MRE11 enrichment at 3 h could be adequately predicted for an independent test dataset with *r* = 0.78 and 0.64, respectively (Fig. 4h,i). Using solely epigenetic information led to comparable levels of performance with *r* = 0.75 for dCas9 and 0.59 for MRE11 (Fig. 4j,k). However, using both mismatch and epigenetic information greatly improved prediction, resulting in *r* = 0.86 for dCas9 and 0.83 for MRE11 (Fig. 4l,m). Comparable levels of predictive power were also achieved for the 30 min timepoint (Extended Data Fig. 2d–i). These results highlight the importance of local epigenetic state in modulating Cas9 activity and provide further evidence that combining epigenetic with mismatch information improves the prediction of genome editing activity^21,43^.

### Increase in chromatin accessibility at Cas9-induced DSBs

**Fig. 5:**
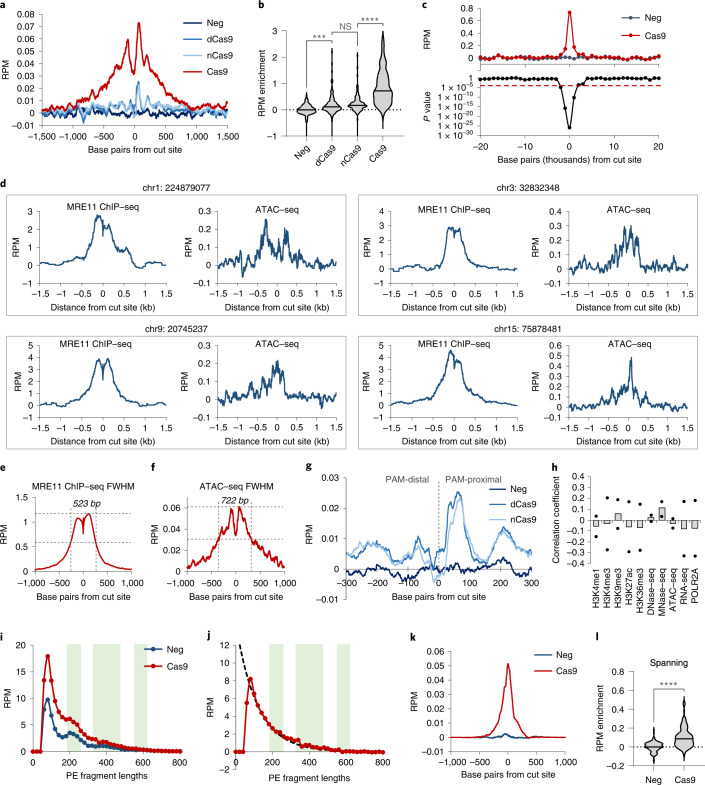
Chromatin accessibility change after DNA damage. **a**, Averaged background-subtracted ATAC–seq profiles across all on-target sites for cells without Cas9 (‘neg’, dark blue); or 3 h after Cas9/‘GG’-gRNA (‘Cas9’, red), dCas9/’GG’ (‘dCas9’, medium blue) or D10A Cas9 nickase/’GG’ delivery (‘nCas9’, light blue). ‘Background-subtracted’ enrichment was obtained by subtracting the number of reads at each position for Cas9-negative cells from Cas9-exposed cells, yielding enrichment values that quantify ‘excess’ accessibility due to Cas9 exposure. **b**, Violin plots of background-subtracted ATAC–seq enrichment (in RPM) at each target site for samples from **a**. Comparison using two-sided unadjusted Student’s *t*-test. ***P < 0.001, ****P < 0.0001. *P* values from left to right are: 0.00027, 0.072 and 8.77 × 10^−14^. **c**, Top: average background-subtracted ATAC–seq enrichment (RPM) in 1 kb windows moving upstream and downstream of all cut sites, for cells without Cas9 (neg), or 3 h after Cas9/‘GG’–gRNA delivery (Cas9). Bottom: two-sided Student’s *t*-test *P* values of difference in enrichment between ‘neg’ and ‘Cas9’ samples in each 1 kb window. **d**, MRE11 ChIP–seq and background-subtracted ATAC–seq signals at four representative cut sites. The genomic coordinate (hg38) of the cut sites are displayed on top of each panel. **e**,**f**, Measurement of FWHM for MRE11 ChIP–seq enrichment (523 bp) versus MRE11 enrichment (722 bp) averaged across all on-target cut sites. **g**, Close-up of averaged background-subtracted ATAC-seq profiles across all on-target sites for cells without Cas9 (‘neg’, darkest blue), dCas9 (‘dCas9’, medium blue) and D10A Cas9 nickase (‘nCas9’, light blue). Zoomed view of **a**. **h**, Correlation between ten epigenetic markers and MRE11-normalized excess ATAC–seq enrichment due to Cas9-mediated DNA damage. *n* = 2 biologically independent experiments. **i**, Histogram of ATAC–seq DNA lengths for cells without Cas9 (‘neg’, blue), or 3 h after Cas9/‘GG’-gRNA delivery (‘Cas9’, red). **j**, Subtraction of the ‘Cas9’ sample by ‘neg’ sample from **j**. Fitting (black dotted curve) was performed using an exponential decay model. **k**,**l**, Same as **a** and **b**, for cells without Cas9 (‘neg’, dark blue) or 3 h after Cas9/‘GG’–gRNA delivery (‘Cas9’, red), but only measuring the subset of ATAC–seq reads that span the cut site. Comparison using two-sided unadjusted Student’s *t*-test. ****P < 0.0001. *P* value is 1.44 × 10^−18^. Source numerical data are available in source data. Source data

It has been proposed that local chromatin decompaction occurs after DNA damage to facilitate repair, but direct evidence has not been observed at single Cas9 DSBs^44,45^. To measure chromatin accessibility changes after DNA damage, we performed ATAC–seq^46^ with and without exposure to Cas9/mgRNA. Averaged background-subtracted ATAC–seq enrichment centred at Cas9 target sites exhibited locally increased accessibility after 3 h of Cas9 exposure (Fig. 5a,b). Excess chromatin accessibility was only detected within 1–2 kb from the cut site (*P* < 9 × 10^−5^) (Fig. 5c). The average full width at half maximum (FWHM) of ATAC–seq chromatin accessibility increase was slightly greater than that of MRE11 (722 bp versus 523 bp, respectively) (Fig. 5d–f). ATAC signal obtained using dCas9 or the D10A Cas9 nickase was much smaller in width and amplitude (Fig. 5a,b,g), suggesting that the large change in chromatin accessibility is specific to Cas9-generated DSB. There was no clear correlation between MRE11-normalized ATAC–seq enrichment and any epigenetic marker (Fig. 5h), suggesting that chromatin opening after Cas9 cleavage occurs independent of chromatin context.

Next, we inferred the lengths of all PE ATAC–seq reads within 1.5 kb from expected target sites. For cells without Cas9, the distribution of sequencing read lengths showed a local maximum that corresponded to nucleosome occupancy footprinting^46^ (Fig. 5i). Cells exposed to Cas9 had excess ATAC–seq reads; the length distribution of the excess reads lacked the nucleosomal footprinting signature and was well fit by an exponential decay, consistent with distances between adjacent Tn5 transposition events that are assumed to be a Poisson point process (Fig. 5j). Assuming nucleosome spacing length of around 200 bp, this implies that the up to 2 kb accessible region from Fig. 5c lost up to ten nucleosomes^47,48^. We further uncovered a subpopulation of ATAC–seq reads spanning the target sites that significantly increased after Cas9 delivery (*P* = 1.44 × 10^−18^, Student’s *t*-test) (Fig. 5k,l), which must correspond to post-cleavage DNA that has undergone ligation and suggests that chromatin recompaction does not occur immediately after ligation. In conclusion, Cas9 cleavage induces a localized, nucleosome-depleted, kilobase-scale region of increased accessibility that can persist after DNA ligation, which potentially facilitates the binding of DNA damage-associated proteins such as repair factors, cohesin and transcription factors to promote successful repair^49–51^.

### Chromatin accessibility dynamics in DSB repair

**Fig. 6:**
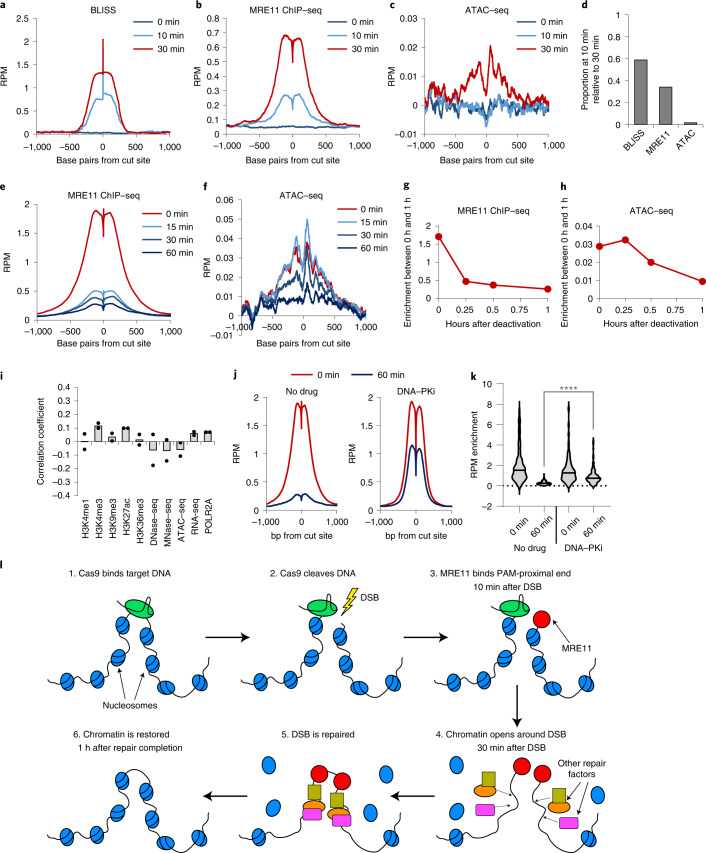
Timescales of DDR recruitment and dissolution. **a**–**c**, Average BLISS (**a**), MRE11 ChIP–seq (**b**), and ATAC-seq (**c**) enrichment across all on-target sites using Cas9 in complex with vfCRISPR ‘GG’ gRNA. Samples were evaluated at 0 min (no Cas9 activation), 10 min and 30 min after Cas9 activation using light. **d**, Quantification of **a**–**c**. Proportion, at 10 min, of the maximal enrichment at 30 min after Cas9 activation. **e**,**f**, Average MRE11 ChIP–seq (**e**) and ATAC–seq (**f**) enrichment across all on-target sites using Cas9 in complex with ‘GG’ pcRNA. Samples were evaluated at 0 min (no deactivation, 2 h after Cas9 delivery), 15 min, 30 min and 60 min after Cas9 deactivation using light. **g**,**h**, Quantification of **e** and **f**. **i**, Proportional change in background-subtracted MRE11 RPM enrichment at all on-target sites is not correlated with any evaluated epigenetic markers. *n* = 2 biologically independent experiments. **j**, Average MRE11 ChIP–seq enrichment across all on-target sites using Cas9 in complex with ‘GG’ pcRNA, evaluated at 0 min (no deactivation) or 60 min after Cas9 deactivation using light, with (right) or without (left) DNA–PKcs inhibitor KU-60648. **k**, Quantification of **j**. Comparison using two-sided unadjusted Student’s *t*-test. ****P < 0.0001. *P* value is 1.45 × 10^−8^. Source numerical data are available in source data. **l**, Cartoon summarizing our findings on DDR in the context of Cas9 cleavage. Within ~10 min after Cas9 cleavage, MRE11 is recruited preferentially to the PAM-proximal side. Approximately 30 min after Cas9 cleavage, chromatin undergoes decompaction around the cut site, potentially to facilitate the recruitment of additional DNA repair factors. Once the DSB is repaired, chromatin accessibility returns to the original pre-DSB state. Source data

The temporal sequence of events after Cas9 cleavage has not been well characterized but can be explored using the very fast light-activatable CRISPR (vfCRISPR) based on a photocaged gRNA (cgRNA)^13^. We delivered Cas9 with the multi-target ‘GG’ cgRNA to HEK293T cells, waited 12 h for stable Cas9 binding, then light-activated Cas9 and performed time-resolved BLISS, MRE11 ChIP–seq and ATAC–seq. DSBs and MRE11 damage responses were undetectable before light activation, confirming that Cas9 is inactive without light exposure (Fig. 6a,b). As early as 10 min after activation, BLISS exhibited the strongest relative enrichment increase followed by MRE11 ChIP–seq signal (Fig. 6a,b), consistent with initial DSB induction followed by repair protein recruitment. ATAC–seq enrichment increased by 30 min after Cas9 activation but not 10 min (Fig. 6c,d and Extended Data Fig. 2j), suggesting that DSB-induced increase in accessibility occurs downstream of initial repair protein recruitment.

After repair of DNA damage, the duration of accessibility increase remains unknown. However, without an effective method for CRISPR deactivation, intracellular Cas9 will repeatedly cleave repaired loci and preclude measurements of chromatin restoration^52^. We therefore employed a light-deactivatable Cas9 based on a photocleavable gRNA (pcRNA) to synchronize Cas9 deactivation, facilitating chromatin profiling through repair completion^52^. We delivered Cas9 with multi-target ‘GG’ pcRNA to HEK293T cells, deactivated Cas9 after 2 h and performed time-resolved MRE11 ChIP–seq and ATAC–seq. After Cas9 deactivation, MRE11 enrichment rapidly declined across all target sites with 75% reduction in enrichment within the first 15 min (Fig. 6e), which probably corresponds to completion of DNA repair^13^. In contrast, the level of chromatin accessibility increase persisted for the first 15 min before declining (Fig. 6f–h), consistent with our previous results in Fig. 5k,l and suggesting that accessibility reversal is delayed compared with MRE11 departure. There was no detectable correlation between MRE11 departure and the tested epigenetic markers (Fig. 6i). Inhibition of DNA–PKcs using KU-60648 prevented MRE11 departure^52^, suggesting that the repair events are dependent on non-homologous end-joining (Fig. 6j–k)^14^.

Our findings on Cas9 activity and DDR are summarized in Fig. 6l. After binding and cleavage of target DNA, Cas9 quickly releases the DNA preferentially from the PAM-proximal side, enabling binding of MRE11 to this DNA end within 10 min. Within 30 min of DSB, chromatin undergoes decompaction whereby nucleosomes ~1 kb from the cut site are evicted, potentially facilitating recruitment of additional DNA repair factors. Once the lesion has been repaired, the nucleosomes are repositioned around the cut site, restoring the chromatin accessibility landscape to pre-cleavage levels.

## Discussion

**Fig. 7:**
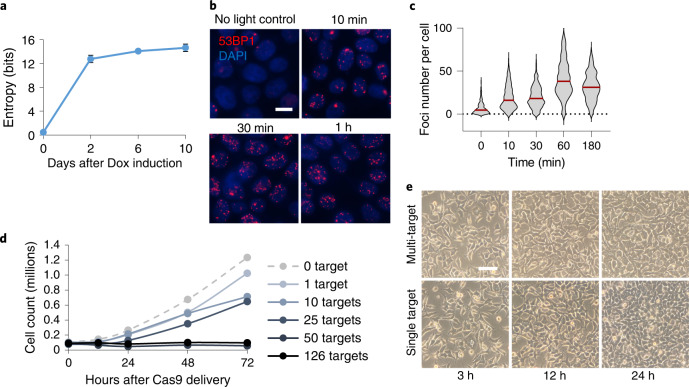
Quantification of entropy and DNA damage generated by mgRNAs. **a**, Cumulative Shannon entropy for the ten-target mgRNAs across the eight on-target sites with detectable indels. Higher entropy corresponds to higher mutation diversity that is necessary for effective cellular barcoding. *n* = 3 biologically independent experiments, data presented as mean ± s.d. **b**, Representative images of 53BP1 immunofluorescence microscopy as a function of time after ‘GG’ multi-target Cas9 activation in HEK293T cells using the light-inducible vfCRISPR system. Cas9/gRNA was electroporated into cells 12 h before light-based Cas9 activation. Scale bar, 10 μm. *n* > 300 cells examined over three independent experiments. **c**, Quantification of number of 53BP1 foci in each cell from **b** at all evaluated timepoints. **d**, Cell counts over time (12 h, 24 h, 48 h and 72 h) after delivery of Cas9 into HEK293T cells with mgRNA targeting various numbers of expected genome-wide sites (0, 1, 10, 25, 50 and 126 expected sites). Data points are the average of two biologically independent samples. **e**, Representative microscopy images of HEK293T cells at different timepoints (3 h, 12 h and 24 h) after electroporation of Cas9 with multi-target (‘GG’) versus single-target (targeting *ACTB*) gRNA. Scale bar, 50 μm. Source numerical data are available in source data. Source data

We report the discovery and applications of multiplexed CRISPR using mgRNAs. We identified tens of thousands of mgRNAs that each target 2 to over 1,000 positions across multiple genomes, providing an extensive resource for rapid adoption. We then combined mgRNAs with high-throughput sequencing readouts to provide the most comprehensive study thus far of Cas9 genome editing and ensuing DDRs at endogenous loci (Supplementary Table 1). The large number and diversity of target sites enables generalizable observations such as the destabilizing impact of even one PAM-distal mismatch on Cas9 binding and better cleavage by bound Cas9 near transcribed regions. Aggregating data across multiple target sites boosts readout signal, allowing us to use ATAC–seq reads across all target sites to measure local nucleosome depletion after Cas9 DNA damage. Furthermore, compatibility with very fast CRISPR activation and deactivation^13,52^ allowed quantification of the dynamics of chromatin accessibility change during and after DNA repair with high temporal resolution. Cas9 with mgRNAs also exhibits advantages over ‘multi-target’ meganucleases^44^ including programmable target positioning, precise time control using CRISPR activation and deactivation, facile delivery without need to generate a stable cell line, and relevance to CRISPR genome editing. Finally, the ability to read mutational outcomes of mgRNA paves the way towards its use as a genetic barcoding tool. Supporting this claim, the indels at eight target sites generated by the ten target mgRNA in HeLa cells demonstrated high barcoding diversity as measured by Shannon entropy^53^ (Fig. 7a).

Our study is not without limitations. First, the mgRNA model system may not translate to native DSBs or single-targeting Cas9. However, this is unlikely given that most of our findings are corroborated with existing literature. Second, our assumption that every mgRNA target site is independent could be challenged if Cas9 binding/cleavage events physically influence measurements at adjacent target sites. However, the median distance between adjacent binding sites (265 kb) and adjacent on-target sites (13.2 Mb) (Fig. 2e) is orders of magnitude greater than the ~2 kb window used for the bulk of analysis, so the effect of nearby off-target Cas9 activity is probably minor. Third, bulk sequencing cannot deconvolute heterogeneity between individual cells, which may be overcome by combining mgRNAs with single-cell imaging^13^ or sequencing readouts^54^. Finally, mgRNA can generate high numbers of simultaneous DSBs in each cell, averaging under 50 per cell for a 126-targeting mgRNA based on the number of 53BP1 foci in immunofluorescence microscopy (Fig. 7b,c). A single DSB delayed cell division, consistent with a previous report^55^, and 50 DSBs blocked cell division (Fig. 7d). We believe the high DSB count is unlikely to influence our results because all experiments were conducted within 3 h of Cas9 delivery during which no altered cellular phenotypes were observed (Fig. 7e and Extended Data Fig. 2j), and relative Cas9 kinetics between different target sites are probably unaffected by the high mutation load.

In conclusion, we developed mgRNAs as an approach to multiplex CRISPR–Cas9 at endogenous sites. Using mgRNAs, we revealed insights on Cas9 target recognition and cleavage activity, and determined the dynamics of chromatin accessibility during repair of Cas9-induced DSBs. We envision that mgRNAs will be a powerful tool to further advance our understanding of CRISPR technologies and DNA repair processes.

## Methods

### SpCas9 purification

SpCas9 purification was done using BL21-CodonPlus (DE3)-RIL competent cells (Agilent Technologies 230245) that were transformed with Cas9 plasmid (Addgene, #67881). Bacteria were grown in 1 L of LB medium, induced with isopropyl-β-d-thiogalactoside overnight and then lysed. The supernatant was clarified and then purified using Ni-NTA beads. A detailed description can be found in ref. ^56^.

### Cell culture

HEK293T cells (ATCC® CRL-3216) and HeLa (ATCC CCL-2) cells were cultured at 37 °C under 5% CO_2_ in Dulbecco’s modified Eagle’s medium (DMEM, Corning) supplemented with 10% FBS (Clontech), 100 units/mL penicillin and 100 µg ml^−1^ streptomycin (DMEM complete). Cells were tested every month for mycoplasma.

A human iPSC, WTC11 cell line^57^ was used for all iPSC experiments in this study. We followed the guidelines of Johns Hopkins Medical Institute for the use of this human iPSC line. Briefly, frozen WTC11 cells were first thawed in 37 °C water bath and washed in Essential 8 Medium (E8; Thermo Fisher Scientific, #A1517001) by centrifugation. After resuspension, WTC cells were plated onto a 6 cm cell culture dish pre-coated with human embryonic cell-qualified Matrigel (1:100 dilution, Corning, #354277). Plate coating should be performed for at least 2 h. Subsequently, 10 µM ROCK inhibitor (Y-27632; STEMCELL, #72308) was supplemented into the E8 medium to promote cell growth and survival. For subculture, WTC11 cells were dissociated from the plate using accutase (Sigma, #A6964) and passaged every 2 days. WTC11 cells were maintained in an incubator at 37 °C with 5% CO_2_.

### Electroporation of Cas9 ribonucleoprotein

A Cas9:mgRNA ribonucleprotein was assembled and electroporated into HEK293T or WTC-11 iPSC cells using 4D-Nucleofector Kits (Lonza, SF Cell Line kit for HEK293 and P3 Primary Cell kit for WTC11) following the manufacturer’s instruction. Oligos used for *trans*-activating CRISPR RNA (tracrRNA) and CRISPR RNAs (crRNAs) are presented in Supplementary Table 2. More details can be found in ref. ^56^.

### Chromatin immunoprecipitation sequencing

The ChIP protocol was adapted from previous literature^28^. Oligonucleotide sequences for library preparation are in Supplementary Table 3. A detailed protocol can be found in ref. ^56^. Briefly, protein A beads were washed twice using BSA buffer and incubated with the antibody for 1–3 h with rotation. Bead–antibody mixtures were washed twice with BSA buffer right before ChIP. Cells were collected and fixed with formaldehyde (1% final) at room temperature. The reaction was quenched using glycine (130 mM final). Cells were then lysed sequentially using three different buffers, sonicated and spun down. The supernatant was collected, and the bead–antibody mixture was added. The ChIP reaction incubated overnight. Bead mixtures were then washed on a magnet seven times, resuspended in reverse crosslink buffer and incubated at 65 °C for 6+ hours. After proteinase K and RNAse A treatments, the DNA was column purified. To prepare ChIP–seq libraries, we performed end repair/dA-tailing reaction, followed by adapter ligation and PCR using PE_i5 and PE_i7XX primer pairs. Final DNA was purified using AMPure beads, quantified via Qubit, pooled and sequenced on a NextSeq 500 (Illumina).

### Genome-wide DSB detection with BLISS

The BLISS protocol was adapted from previous literature^39^. All oligonucleotide sequences are provided in Supplementary Table 4. A detailed protocol can be found in ref. ^56^. In short, BLISS adapters were annealed and phosphorylated RA3 oligonucleotides were adenylated. In total, 400,000 cells were seeded into a 24-well plate for each reaction, washed once with PBS, fixed with 4% paraformaldehyde for 10 min, then washed three times with PBS. Cells were then subjected to a first round of lysis, followed by a PBS wash, a second round of lysis and two PBS washes. Cells were then washed twice with CutSmart Buffer (NEB), and subjected to DNA end-blunting reaction. Cells were then washed twice with CutSmart Buffer followed by adenylation of DNA ends. Cells were washed twice with CutSmart Buffer and with T4 Ligase Buffer, followed by in situ adapter ligation. Samples were then washed four times with high-salt buffer to remove unligated adapters. DNA was extracted by adding extraction buffer and proteinase K, incubating at 55 °C overnight and column purifying DNA the day after. DNA was then sonicated, in vitro transcribed and purified. RA3 adapter was ligated to the purified RNA, and the product was purified. Samples were reverse transcribed and PCR amplified, and the final DNA was purified using AMPure beads. Samples were pooled, quantified with QuBit, Bioanalyzer and qPCR, then sequenced on a NextSeq 500 using high-output paired sequencing, with 64 bp for read 1 and 36 bp for read 2. Only the subset of reads with the correctly matching 13 bp constant adapter region (CGCCATCACGCCT) in read 1 was used for subsequent analysis.

### Measurements of mutations at mgRNA targets

A PiggyBac system was used to transpose HeLa cells with a vector carrying Cas9 under the control of a Tet-On inducible promoter and a puromycin resistance gene. Two days after transposition, clonal cell lines were isolated and grown in presence of 2 μg ml^−1^ of puromycin. Vectors carrying 10-target or 20-target mgRNAs were made by cloning forward and reverse mgRNA oligos (carrying respectively a 5′-CACCG and a 5′-CAAA and 3′-C overhang; Supplementary Table 5) into the LentiGuide-Hygro plasmid (Addgene #139462). Plasmid was digested using BsmBI-v2 (NEB, #R0739), gel-extracted and then ligated overnight with the pre-annealed phosphorylated forward and reverse mgRNA oligos. Cells (NEB, #C2987) were transformed with the ligation product and plated following the manufacturer’s instructions. The following day, individual colonies were selected and grown in selection media; plasmids were purified the next day using QIAprep Kit (Qiagen, #27106). Correct insertion of the mgRNA was verified via Sanger sequencing. For lentivirus production, Lenti-X 293T cells (takarabio, #632180) were grown in 10 cm dishes up to ~70% confluency. Then, 5.25 μg of transfer plasmid was mixed with 0.75 μg of pMD2.G (Addgene, #12259) and 1 μg of psPAX2 (Addgene, #12260), and with 21 μl of TransIT-Lenti (Mirus, #6603). The mixture was incubated for ~15 min and added dropwise to the cells. The viral supernatant was collected at 36 h, 48 h and 60 h, and filtered and concentrated using Lenti-X Concentrator (takarabio, #631232), according to the manufacturer’s instructions. Doxycycline (Dox)-inducible Cas9 monoclonal cells were grown to ~60 % confluency in six-well plates. Cells were exposed to virus carrying mgRNA (~0.3–0.5 multiplicity of infection) and 8 μg ml^−1^ polybrene for 24 h. Two days after infection, cells were exposed to 100 μg ml^−1^ hygromycin and kept under such selection conditions for all subsequent experiments. Death of half of the cells confirmed successful plasmid integration at the estimated multiplicity of infection. An initial set of stably transduced cells were collected before Dox addition as timepoint zero. Cells were then grown in 24-well plates under exposure to 2 μg ml^−1^ of Dox. At different timepoints after induction, a number of cells were collected during passaging and their gDNA was extracted. For the ten-target mgRNA, a no-Dox control experiment was performed in parallel.

gDNA was extracted from using Qiagen DNeasy kit (Qiagen, #69506), eluted in 60 μl of elution buffer and quantified using QuBit (Thermo). One nanogram of gDNA was amplified via three PCRs: two nested PCRs to amplify the target region and a third, indexing PCR to attach the NGS adapters and indices. PCR-1 was run to 20 cycles using the primers presented in Supplementary Table 6. One microlitre of 1:10 dilution of unpurified PCR-1 product was used for PRC-2, which was run to 20 cycles using the primers presented in Supplementary Table 7. The PCR-2 product was purified using 1× volume of AMPure XP beads (Beckman Coulter) and eluted in 15 μl of IDTE buffer (IDT DNA). One microlitre of this product was used for PCR-3, which was run to seven cycles using the primers from Supplementary Table 8. The final product was purified using 0.8× volume of AMPure XP beads, eluted in 15 μl of IDTE and quantified using QuBit. Products from different samples were pooled and sequenced using a MiSeq (Illumina). We found conditions for pooling primers from different targets that yielded a balanced representation of all the sequenced targets among the NGS reads. For the ten-target mgRNA, we pooled all the PCR-1 primers and all the PCR-2 primers in equimolar amounts to a final concentration of 5 μM per oligo. For the 20-target mgRNA, we made three sets of primers per PCR: set 1 with targets 2–6, set 2 with targets 8–11 and set 3 with targets 1, 7 and 12. Targets were then de-multiplexed during the data analysis (see below).

### Determining mutation levels and mutation outcomes of mgRNAs

To determine the mutation levels of the different mgRNA targets, we first de-multiplexed these targets (which were amplified in a multiplexed fashion) by aligning the first 50 bp of each PE read to the genome. A given read was considered to contain an mgRNA target if the PE alignment fell within a window of 1,000 bp from the expected genomic location of the target. A mutation was called if the intact theoretical protospacer sequence was not found in the read.

For classification of the mgRNA target mutations, we defined for each target site two key sequences that were, respectively, 20 bp upstream and downstream of the expected genomic location of the cut site. For each read aligning to a target site, these two key sequences were identified and the distance between them was computed. Reads with distances shorter than the expected value were classified as deletions, while reads with distances longer than expected were classified as deletions. Reads with the expected distance between the key sequences but with mutations in the protospacer were classified as single-nucleotide variants (SNVs).

### ATAC–seq

ATAC–seq was performed following the Omni-ATAC protocol^58^ using the amplification protocol and primers described in ref. ^59^. Primers are also presented in Supplementary Table 9. A detailed protocol can be found in ref. ^56^. Cells were washed with PBS, collected via scraping and counted. A total of 50,000 cells were used for ATAC. Collected cells were then pelleted, the supernatant was removed and the cells were resuspended in 50 µl of cold lysis buffer, gently mixed and incubated on ice for 3 min. One millilitre of wash buffer was then added and gently mixed. Nuclei were then pelleted, resuspended in 50 µl of transposition reaction and incubated at 37 °C for 30 min. Transposed DNA was column purified and eluted in 21 µl of EB. Samples were pre-amplified, followed by qPCR to determine the number of cycles needed for final amplification (one-third of saturation). Final DNA was purified using AMPure beads and eluted in 32 μl IDTE. Final libraries were quantified using 2% agarose gel, pooled, quantified with QuBit, Bioanalyzer and qPCR, then sequenced on a NovaSeq 500 (Illumina) using paired 2 × 50 bp reads.

### CRISPR activation and deactivation

The special cgRNA or pcRNAs were used in the place of normal crRNAs when complexed with tracrRNA. For activation, Cas9/cgRNA was first electroporated into cells, plated onto 12-well plates, then incubated for 12 h to allow stable Cas9 binding but not cleavage. Next, cells were exposed to 1 min of 365 nm light exposure from a handheld blacklight (https://www.amazon.com/JAXMAN-Ultraviolet-365nm-Detector-Flashlight/dp/B06XW7S1CS/). Either one, three or six flashlights were used at once. When multiple flashlights are used, they are conveniently held together using a 3D-printed flashlight holder. (https://github.com/rogerzou/chipseq_pcRNA/blob/master/Jaxman_LED_flashlight_holder_design/files/8zeFECPViSo.stl). Samples were collected without light exposure, or 10 m and 30 m after light exposure.

For deactivation, Cas9/pcRNA was first electroporated into cells, plated onto 12-well plates, incubated for 2 h, then exposed to light of the same dose. Samples were collected during the time of light exposure, or at 1 h, 2 h and 4 h after light exposure.

### Immunofluorescence microscopy of 53BP1 foci after multi-target Cas9 activation

The number of endogenous 53BP1 foci in cells was evaluated through immunofluorescence microscopy. One hour after Cas9:cgRNA electroporation, we illuminated the cell samples with 365 nm light for 30 s to trigger Cas9 cleavage. The samples were fixed with 4% of paraformaldehyde in PBS for 10 min at different times (0 min, 10 min, 30 min, 1 h and 3 h) and quenched with glycine in PBS (final of 0.1 M) for 10 min. After rinsing with PBS, 0.5% Triton-X was used to permeabilize cell membrane for 10 min. To passivate the sample for 1 h at room temperature, 2% w/v BSA in PBS was used. Anti-53BP1 antibody (Novus Biological, NB100-304) was diluted 1:1,000 in PBS and added into the chamber. After 1 h incubation, primary antibody was removed and the sample was washed three times with PBS. Alexa647 (Thermo Fisher Scientific, A-21235) conjugated secondary antibody was diluted in 1:1,000 and applied to the sample for 1 h. Finally, the sample was rinsed three times and mounted with Prolong Diamond mounting medium (Thermo Fisher Scientific) overnight. We imaged all cell samples using Nikon Ti-E fluorescence microscope equipped with Hamamatsu CMOS camera and an objective of 40× magnification. Cell samples were scanned in *z*-stack with a total depth of 5 μm such that all 53BP1 foci within the cell nuclei (DAPI) were captured. Three-dimensional image datasets were first processed into 2D datasets in FIJI using maximum intensity projection. The number of 53BP1 foci per nuclei was analysed with a custom-built CellProfiler3 pipeline.

### Discovery and characterization of mgRNA sequences

Starting from a 280 bp SINE sequence, for all 20 bp substrings in both the forward the reverse complement direction, we obtained all 20 bp sequences with up to three mismatches from template restricted to the nine most PAM-proximal nucleotides. GC content was restricted to 40–70%. This resulted in 75,626 unique target sequences. To determine the number alignments for each target, we outputted each gRNA + PAM into a FASTA file and ran bowtie2 with ‘-k 1000’ mode, which searches up to 1,000 alignments for each line in the FASTA, that is, each target sequence.

*bowtie2 -k 1000 -f -x [path to genome] -U [path to input FASTA file] -S [path to output SAM file]*

We iterated through all alignments (up to 1,000) for each gRNA, then determined whether each alignment was within a RefSeq gene annotation and the ChromHMM epigenetic labelling^60^. As HEK293T ChromHMM was not available, we curated ChromHMM annotations from A549 (E114), GM12878 (E116), HeLa-S3 (E117) and K562 (E123), and the final ChromHMM annotation for each target was the consensus of these four annotations. Annotation data were obtained from https://egg2.wustl.edu/roadmap/web_portal/index.html.

### Ambiguous read proportions from simulated ChIP–seq reads

For gRNA with 100–300 on-target sites in the genome, we simulated 100 PE 200–600-bp-long (uniform distribution) sequencing reads. The reads were randomly chosen to either span the cut site, reside PAM-distal or reside PAM-proximal to the cut. For PAM-distal or PAM-proximal reads, the distance from the edge of the DNA to the cut site was drawn from an exponential distribution. Both 2 × 36 PE reads and 2 × 75 PE reads were simulated.

The PE reads were outputted to FASTA files (read 1 and read 2), and bowtie2 was used to determine up to ten alignments for each simulated read pair:

*bowtie2 -f -p 9 –local -k 10 -X 1000 –no-mixed –no-discordant -x [path to genome] -1 [path to read1] -2 [path to read2] -S [path to output SAM]*

The code subsequently determines whether the original position of the read pairs matches the best alignment based on bowtie2, and whether this best alignment has the uniquely best alignment score. The proportion of reads that satisfy these requirements represent the proportion of uniquely best alignments. The proportion of ambiguous alignments is 1 minus this value.

### Ambiguous read proportions from real ChIP–seq reads

We used all dCas9 binding positions for analysis. For each binding position, we converted PE ChIP–seq reads found within a specified window width centred at the Cas9 binding site into FASTA read 1 and read 2 file formats. Then the section ‘Ambiguous read proportions from simulated ChIP–seq reads’ was followed, starting with use of bowtie2. Window widths of 1500 bp were used for Cas9 ChIP–seq, and 2,500 bp for MRE11.

### Nucleotide composition analysis of region surrounding gRNA on-target sites

The local genomic sequences for each expected on-target site for ‘CT’, ‘GG’ and ‘TA’ gRNAs were obtained, then aligned by the Cas9 cut site (PAM oriented downstream of the cut). At each base-pair position relative to the cut site, the nucleotide was tallied and/or displayed. This analysis was performed ±500 bp from cut sites.

### General data pre-processing for ChIP–seq, BLISS and ATAC–seq

Reads were demultiplexed after sequencing using bcl2fastq. PE reads were aligned to hg19 or hg38 using bowtie2. Samtools was used to filter for mapping quality ≥25, remove singleton reads, convert to BAM format, remove potential PCR duplicates and index reads.

### Calculating enrichment for MRE11, Cas9, γH2AX and 53BP1 ChIP–seq

We determined the reads per million (RPM) in specific window widths centred at all cut sites. We used a window of 200 kb for both 53BP1 and γH2AX, 2,500 bp for MRE11 and 1,500 bp for Cas9. For MRE11 and Cas9, additional code analyses the exact read positions and determines if a PE sequencing read fragment spans the cut site (‘span’), or if a sequenced DNA fragment begins within 5 bp from the cut site (‘abut’). To determine ‘dist + 4’, ‘dist − 4’, ‘prox + 4’, or ‘prox − 4’, we analysed the DNA fragment position according to the rules specified for these read species.

### Enrichment profiles for MRE11 and Cas9 ChIP–seq (also spanning ATAC–seq) at base-pair resolution

At each genomic position in a window centred at each cut site, each PE read within this window is retrieved. The number of PE reads that map to each base pair is tallied. The middle region of PE read fragment that is not likely to be sequenced is also included in this tally. We used a window of 2,500 bp for MRE11, 1,500 bp for Cas9 and 3 kb for ATAC–seq.

### Enrichment profiles for γH2AX, 53BP1 and ATAC–seq at window widths

To obtain profiles of γH2AX and 53BP1, we calculated the number of sequencing reads (RPM) in each 10 kb window from the cut site, extending to 2 mb both upstream and downstream of cut sites. For ATAC–seq, we calculated RPM in a 4 bp sliding window incremented every 1 bp, extending to 1.5 kb both upstream and downstream of cut sites.

To determine wider levels of potential ATAC-seq enrichment, we used the same function to calculate RPM in each 1 kb window from the cut site, extending to 50 kb both upstream and downstream of cut sites.

### Genome-wide Cas9 binding from dCas9 ChIP–seq

We used macs2 to find all dCas9 binding peaks, using a no-Cas9 sample for negative control, via the command:

*macs2 callpeak -t [path/to/sample] -c [path/to/negctrl]–outdir [path/to/output] --name [name/of/output] -f BAMPE -g hs*

Next, for each macs2 discovered peak with fold enrichment ≥4, a custom algorithm attempts to identify the target sequence position for Cas9 binding or cleavage that best explains the peak. This may be problematic for target sites with multiple mismatches. We use the following assumption to simplify the problem: (1) there is only one correct Cas9 binding/cleavage sequence within the 400 bp window of the macs2-predicted peak centre, and (2) the correct Cas9 binding/cleavage sequence is one with the fewest mismatches.

### Enrichment measurements of epigenetic markers

Datasets used are indicated in ‘Data availability’. For enrichment, we use a 50 kb radius for RNA-seq, H3K4me1, H3K4me3, H3K9me3, H3K27ac and H3K36me3, a 50 bp radius for DNase I and ATAC–seq, and a 10 bp radius for micrococcal nuclease digestion with deep sequencing (MNase–seq). The number of reads that are found in each specified window width is outputted, normalized by the total RPM.

### Machine learning model

We used the random forest regressor from scikit-learn^61^. For mismatch information, features were obtained from one-hot encoding of mismatch state at each position along the protospacer. For epigenetic information, the RPM enrichment was directly used as features. The predicted output is the level of dCas9 binding or MRE11 enrichment, also measured as RPM. The machine learning model was trained using five-fold cross-validation on a training dataset composed of a random 70% of the total dataset. The remaining 30% was used for evaluation and featured in these figures comparing predicted versus actual values.

### ATAC–seq read length distributions

For each PE ATAC–seq read fragment in a 3 kb window centred at all Cas9 on-target sites, its length was recorded. The distribution of DNA length across all target sites, along with exponential decay curve fitting, was computed in Microsoft Excel.

### Statistics and reproducibility

ChIP–seq, ATAC–seq, amplicon sequencing and BLISS experiments were performed in biological replicates. No statistical method was used to pre-determine sample size. No data were excluded from the analyses. The experiments were not randomized. The investigators were not blinded to allocation during experiments and outcome assessment.

### Reporting summary

Further information on research design is available in the Nature Research Reporting Summary linked to this article.

## Online content

Any methods, additional references, Nature Research reporting summaries, source data, extended data, supplementary information, acknowledgements, peer review information; details of author contributions and competing interests; and statements of data and code availability are available at 10.1038/s41556-022-00975-z.

## Supplementary information

Reporting Summary

Supplementary Table 1 Supplementary Tables 1–9.

## Data Availability

Deep-sequencing data generated for this study have been deposited in Sequence Read Archive under BioProject accession PRJNA733683. Sequencing data were analysed using the hg38 genome assembly (https://www.ncbi.nlm.nih.gov/assembly/GCF_000001405.26). Previously published, publicly available epigenetic datasets used in this study are from HEK293 cell lines: ATAC–seq (SRR6418075), DNase I (ENCFF120XFB), H3K4me1 (ENCFF909ESY), H3K4me3 (ENCFF912BYL), H3K9me3 (ENCFF141ZEQ), H3K27ac (ENCFF588KSR), H3K36me3 (ENCFF593SUW), MNase–seq (ERR2403161) and RNA-seq (SRR5627161). Datasets starting with ENCFF can be found and downloaded from ENCODE (https://www.encodeproject.org/). Dataset starting with SRR or ERR can be found and downloaded from NIH’s SRA (https://www.ncbi.nlm.nih.gov/sra). Source data are provided with this paper. All other data supporting the findings of this study are available from the corresponding author on reasonable request.

## Source data

Source Data 1 Source data for Fig. 1.
Source Data 2 Source data for Fig. 2.
Source Data 3 Source data for Fig. 3.
Source Data 4 Source data for Fig. 4.
Source Data 5 Source data for Fig. 5.
Source Data 6 Source data for Fig. 6.
Source Data 7 Source data for Fig. 7.
Source Data 8 Source data for Extended Data Fig. 1.
Source Data 9 Source data for Extended Data Fig. 2.

## Code availability

Analysis code is available on GitHub (https://github.com/rogerzou/multitargetCRISPR). This software is open-source, modular and well documented. It enables in silico discovery and characterization of mgRNAs, alongside comprehensive analysis of ChIP–seq, BLISS and ATAC–seq datasets.
