# Supplementary material for: Massively parallel genomic perturbations with multi-target CRISPR interrogates Cas9 activity and DNA repair at endogenous sites
Source: Nat Cell Biol. 2022 Sep 5;24(9):1433–44. doi: 10.1038/s41556-022-00975-z (PMC9481459; doi:10.1038/s41556-022-00975-z)
Supplement: Supplementary file 1 — Reporting Summary [file 41556_2022_975_MOESM1_ESM.pdf]

nature portfolio

Corresponding author(s): Taekjip Ha

Last updated by author(s): Jun 16, 2022

Reporting Summary

Nature Portfolio wishes to improve the reproducibility of the work that we publish. This form provides structure for consistency and transparency in reporting. For further information on Nature Portfolio policies, see our Editorial Policies and the Editorial Policy Checklist.

Statistics

For all statistical analyses, confirm that the following items are present in the figure legend, table legend, main text, or Methods section.

n/a	Confirmed	
☐	☒	The exact sample size (*n*) for each experimental group/condition, given as a discrete number and unit of measurement
☐	☒	A statement on whether measurements were taken from distinct samples or whether the same sample was measured repeatedly
☐	☒	The statistical test(s) used AND whether they are one- or two-sided<br>*Only common tests should be described solely by name; describe more complex techniques in the Methods section.*
☒	☐	A description of all covariates tested
☐	☒	A description of any assumptions or corrections, such as tests of normality and adjustment for multiple comparisons
☐	☒	A full description of the statistical parameters including central tendency (e.g. means) or other basic estimates (e.g. regression coefficient) AND variation (e.g. standard deviation) or associated estimates of uncertainty (e.g. confidence intervals)
☐	☒	For null hypothesis testing, the test statistic (e.g. *F*, *t*, *r*) with confidence intervals, effect sizes, degrees of freedom and *P* value noted<br>*Give P values as exact values whenever suitable.*
☒	☐	For Bayesian analysis, information on the choice of priors and Markov chain Monte Carlo settings
☒	☐	For hierarchical and complex designs, identification of the appropriate level for tests and full reporting of outcomes
☐	☒	Estimates of effect sizes (e.g. Cohen's *d*, Pearson's *r*), indicating how they were calculated

Our web collection on statistics for biologists contains articles on many of the points above.

Software and code

Policy information about availability of computer code

Data collection	No software was used for data collection
Data analysis	bcl2fastq2, bowtie2, macs2, samtools1.10, scikit-learn1.1, custom code available in https://github.com/rogerzou/multitargetCRISPR

For manuscripts utilizing custom algorithms or software that are central to the research but not yet described in published literature, software must be made available to editors and reviewers. We strongly encourage code deposition in a community repository (e.g. GitHub). See the Nature Portfolio guidelines for submitting code & software for further information.

Data

Policy information about availability of data

All manuscripts must include a data availability statement. This statement should provide the following information, where applicable:

- Accession codes, unique identifiers, or web links for publicly available datasets
- A description of any restrictions on data availability
- For clinical datasets or third party data, please ensure that the statement adheres to our policy

Deep-sequencing data that support the findings of this study have been deposited in Sequence Read Archive under BioProject accession PRJNA733683. Sequencing data was analyzed using the hg38 genome assembly (https://www.ncbi.nlm.nih.gov/assembly/GCF_000001405.26). Previously published, publicly available epigenetic datasets used in this study are from HEK293 cell lines: ATAC-seq (SRR6418075), DNaseI (ENCFF120XFB), H3K4me1 (ENCFF909ESY), H3K4me3 (ENCFF912BYL), H3K9me3 (ENCFF141ZEQ), H3K27ac (ENCFF588KSR), H3K36me3 (ENCFF593SUW), MNase-seq (ERR2403161), and RNA-seq (SRR5627161). Datasets starting with ENCFF can be found and downloaded from ENCODE (https://www.encodeproject.org/). Dataset starting with SRR or ERR can be found and downloaded from NIH's SRA (https://www.ncbi.nlm.nih.gov/sra). Source data have been provided in Source Data. All other data supporting the findings of this study

are available from the corresponding author on reasonable request.
Analysis code is available on GitHub: https://github.com/rogerzou/multitargetCRISPR.

Field-specific reporting

Please select the one below that is the best fit for your research. If you are not sure, read the appropriate sections before making your selection.

☒ Life sciences ☐ Behavioural & social sciences ☐ Ecological, evolutionary & environmental sciences

For a reference copy of the document with all sections, see nature.com/documents/nr-reporting-summary-flat.pdf

Life sciences study design

All studies must disclose on these points even when the disclosure is negative.

Sample size	No statistical method was used to predetermine sample size.
Data exclusions	No data was excluded from the analysis
Replication	Reproducibility was confirmed by computing Pearson correlation between replicates (when applied). This analysis is clearly stated and shown in the text, methods section, or supplementary materials. All experiments were performed in biological replicates.
Randomization	The experiments were not randomized. This is not required for the current study because there is no participants involved in the experiments.
Blinding	The Investigators were not blinded to allocation during experiments and outcome assessment. This is not relevant for this study because there is no participants involved in the experiments.

Reporting for specific materials, systems and methods

We require information from authors about some types of materials, experimental systems and methods used in many studies. Here, indicate whether each material, system or method listed is relevant to your study. If you are not sure if a list item applies to your research, read the appropriate section before selecting a response.

Materials & experimental systems

n/a	Involved in the study	
☐	☒	Antibodies
☐	☒	Eukaryotic cell lines
☒	☐	Palaeontology and archaeology
☒	☐	Animals and other organisms
☒	☐	Human research participants
☒	☐	Clinical data
☒	☐	Dual use research of concern

Methods

n/a	Involved in the study	
☐	☒	ChIP-seq
☒	☐	Flow cytometry
☒	☐	MRI-based neuroimaging

Antibodies

Antibodies used	Cas9 – Diagenode C15310258; MRE11 – Novus NB100-142; γH2AX – Abcam ab81299; 53BP1 – Novus NB100-305; Alexa647 Secondary antibody – Thermofisher, A-21235
Validation	For all the antibodies used, there was a statement in the manufacturer's website indicating that they are suitable for immunoprecipitation assays. Also, we performed a no-treatment (negative) control for all the antibodies and cell lines tested.

Eukaryotic cell lines

Policy information about cell lines

Cell line source(s)	HEK293T (ATCC® CRL-3216™) and HeLa (ATCC® CCL-2™)
Authentication	The cell lines were not authenticated
Mycoplasma contamination	Cells were tested regularly for mycoplasma contamination and tested negative.
Commonly misidentified lines (See ICLAC register)	None

ChIP-seq

Data deposition

☒ Confirm that both raw and final processed data have been deposited in a public database such as GEO.

☒ Confirm that you have deposited or provided access to graph files (e.g. BED files) for the called peaks.

Data access links<br>*May remain private before publication.*	https://www.ncbi.nlm.nih.gov/bioproject/PRJNA733683/
Files in database submission	fastq files of all ChIP-Seq and ATAC-Seq experiments, including biological replicates
Genome browser session<br>(e.g. UCSC)	https://www.ncbi.nlm.nih.gov/bioproject/PRJNA733683/

Methodology

Replicates	Two biological replicates.
Sequencing depth	Paired-end, 2x36 bp, 15 million paired-end reads per sample
Antibodies	Cas9 – Diagenode C15310258; MRE11 – Novus NB100-142; γH2AX – Abcam ab81299; 53BP1 – Novus NB100-305
Peak calling parameters	macs2 callpeak -t [path/to/sample] -c [path/to/negctrl] --outdir [path/to/output] --name [name/of/output] -f BAMPE -g hs<br>For each macs2 discovered peak with fold enrichment >= 4, a custom algorithm (publicly available on github, see above) attempts to identify the target sequence position for Cas9 binding or cleavage that best explains the peak
Data quality	Reads were filtered for mapping quality >= 25. Singleton reads, potential PCR duplicates and index reads were removed.
Software	bowtie2, macs2, samtools<br>custom code available at https://github.com/rogerzou/multitargetCRISPR
